# The Role of Inhibition in a Computational Model of an Auditory Cortical Neuron during the Encoding of Temporal Information

**DOI:** 10.1371/journal.pcbi.1004197

**Published:** 2015-04-16

**Authors:** Daniel Bendor

**Affiliations:** Institute of Behavioural Neuroscience, Department of Experimental Psychology, University College London, London, United Kingdom; Indiana University, United States of America

## Abstract

In auditory cortex, temporal information within a sound is represented by two complementary neural codes: a *temporal representation* based on stimulus-locked firing and a *rate representation*, where discharge rate co-varies with the timing between acoustic events but lacks a stimulus-synchronized response. Using a computational neuronal model, we find that stimulus-locked responses are generated when sound-evoked excitation is combined with strong, delayed inhibition. In contrast to this, a non-synchronized rate representation is generated when the net excitation evoked by the sound is weak, which occurs when excitation is coincident and balanced with inhibition. Using single-unit recordings from awake marmosets (*Callithrix jacchus*), we validate several model predictions, including differences in the temporal fidelity, discharge rates and temporal dynamics of stimulus-evoked responses between neurons with rate and temporal representations. Together these data suggest that feedforward inhibition provides a parsimonious explanation of the neural coding dichotomy observed in auditory cortex.

## Author Summary

How does our auditory system represent time within a sound? Previous work has demonstrated that both the firing rate of neurons (rate code) and the timing of their stimulus-evoked responses (temporal code) can be used by auditory cortical neurons to represent temporal information. We investigated the underlying mechanisms of these two neural representations using a computational model of a cortical neuron. We found that the timing and magnitude of inhibition determined whether neurons responded to an acoustic stimulus using a rate or temporal code. Our model predicts several differences in the response pattern of neurons using rate and temporal representations, which we next validated with electrophysiological data recorded from the auditory cortex of non-human primates. Together these data suggest that feedforward inhibition provides a parsimonious explanation of how rate and temporal representations are generated in auditory cortex.

## Introduction

Temporal processing is fundamentally important for perceiving and discriminating acoustic stimuli [1,2]. Specifically, the timing between successive acoustic events is used by the auditory system in the recognition of musical rhythms [3,4], human speech [5,6] and conspecific vocalizations [7,8]. When acoustic events occur in a sequence, our perception of this sequence depends on the time interval between successive events. When inter-event time intervals are longer than 25 ms, we perceive a stream of discretely occurring sounds, commonly referred to as acoustic flutter [9,10]. At shorter time intervals, this percept changes from flutter to fusion; the sensation of discretized events is lost and the resulting fused percept generally has a pitch equal to the repetition rate of acoustic events [11]. The flutter/fusion perceptual boundary is not unique to the auditory system. An analogous perceptual boundary occurs for both visual stimuli (flicker/fusion) [12] and tactile stimuli (flutter/vibration) [13].

**Fig 1:**
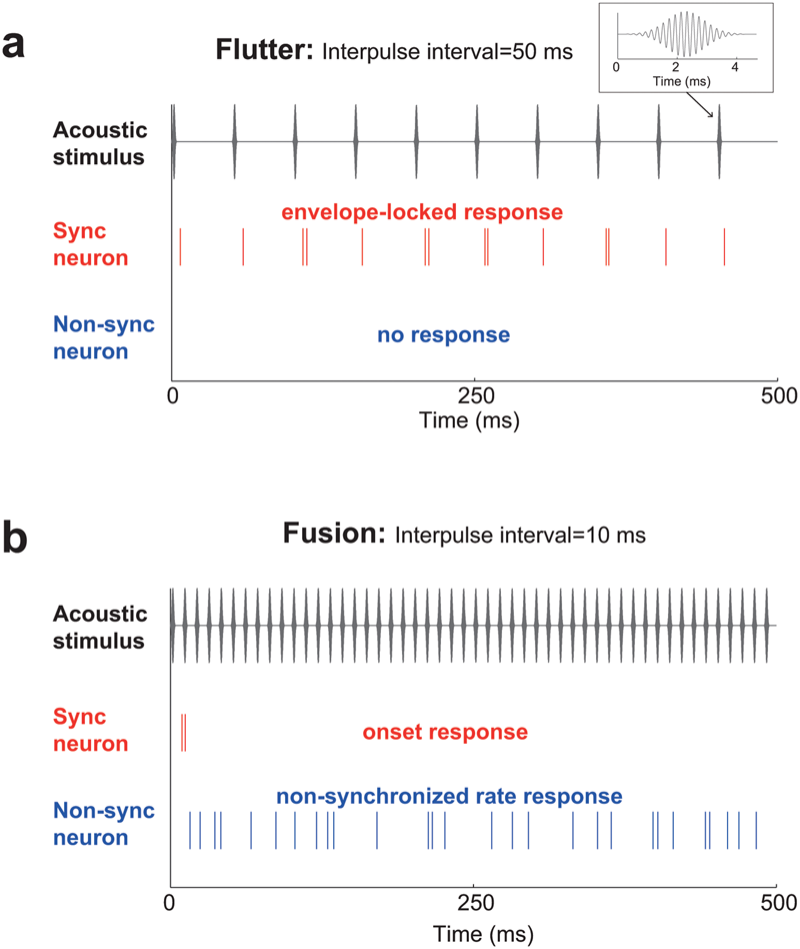
Schematic of synchronized and non-synchronized responses from auditory cortical neurons in response to acoustic pulse trains generating flutter and fusion percepts. Each plot is subdivided (from top to bottom) into an illustration of the acoustic pulse train (gray), and the evoked neural response from synchronized neurons (red) and non-synchronized neurons (blue). The inset plot in (a) shows a single acoustic pulse (5 kHz carrier frequency). a. An acoustic pulse train generating a *flutter* percept (interpulse interval = 50 ms). b. An acoustic pulse train generating a *fusion* percept (interpulse interval = 10 ms).

The dichotomous categorization of a sequence of brief sounds into the perceptions of flutter and fusion is reflected in the corresponding neural representations of these sounds. Within auditory cortex, a sequence of brief sounds, hereinafter referred to as an acoustic pulse train, is encoded with either a temporal or rate representation, for longer and shorter interpulse intervals (IPIs), respectively [14–17]. A temporal representation is provided by neurons with envelope-locked responses, referred to as “synchronized neurons”, reflecting their ability to synchronize their spikes to each acoustic pulse (Fig. 1a). However, the temporal fidelity of this synchronization degrades at shorter IPIs, with an encoding boundary near the flutter/fusion perceptual boundary. In the perceptual range of fusion, synchronized neurons generally elicit only an onset response, and thus cannot be used to discriminate between these shorter IPIs (Fig. 1b). In addition to synchronized responses, neurons can also produce “non-synchronized” responses to acoustic pulse trains [14,17–19]. Non-synchronized neurons increase their firing rate monotonically with decreasing IPIs over the perceptual range of fusion without exhibiting envelope-locked responses (Fig. 1b). While non-synchronized neurons are generally unresponsive at IPIs in the range of flutter (Fig. 1a), the combined neural representations from synchronized and non-synchronized neurons are sufficient to encode temporal information across a wide range of IPIs, spanning the percepts of both flutter and fusion.

This dichotomy between synchronized and non-synchronized responses is not unique to the auditory system; an analogous dichotomy exists in primary visual cortex for representing spatially modulated stimuli [20–22]. When presented with drifting visual gratings, simple cells synchronize their firing to individual bars of the gratings while complex cells produce a non-synchronized discharge pattern. This difference is reflected in the organization of each cell’s receptive field- excitation and inhibition are spatially segregated in simple cells, but spatially overlapping in complex cells. We reasoned that synchronized and non-synchronized responses in auditory cortex could be generated by a similar relationship between excitation and inhibition, with the degree of segregation between these two inputs varying in the time domain, rather than the spatial domain. To investigate this, we simulated an auditory cortical neuron using an integrate-and-fire computational neuronal model [23–24], and measured how changing the relative timing between excitatory and inhibitory inputs affected a neuron’s representation of temporal information.

## Results

**Fig 2:**
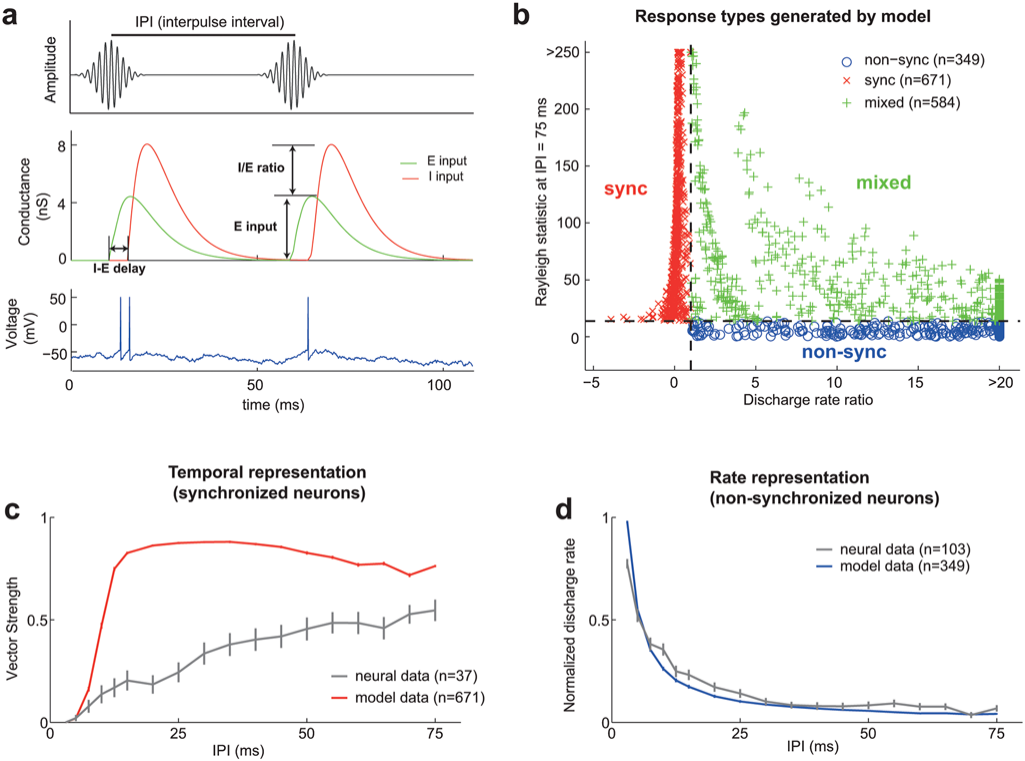
Computational model of an auditory cortical neuron. Error bars indicate SEM. a. The acoustic stimulus (top) used in our neurophysiological experiments was a narrowband acoustic pulse train. Each pulse was converted into an excitatory and inhibitory conductance in our computational model, using an alpha function with a time constant of 5 ms (middle). Three parameters could be altered (I-E delay, E input, and I/E ratio). Above threshold changes in the membrane voltage generated spikes (bottom), which could be further analyzed to measure the response properties of the simulated neuron. b. Classification of neural coding regime based on the two criteria (dashed lines)- y axis: Rayleigh statistic at an IPI of 75 ms>13.8, x axis: Discharge rate ratio>1. Neurons were classified as having a non-synchronized (o), synchronized (x), or mixed (+) response. c. Comparison of stimulus synchronization in real (gray) and simulated (red) synchronized neurons across different IPIs (3–75 ms). d. Comparison of normalized discharge rate in real (gray) and simulated (blue) non-synchronized neurons across different IPIs (3–75 ms).

We developed an integrate-and-fire computational model of an auditory cortical neuron [23], based on previously reported data obtained using in-vivo, whole-cell recordings from rodent primary auditory cortex [24] (see Methods). We tested our model with acoustic pulse trains spanning the perceptual range of flutter/fusion perception, with interpulse intervals (IPIs) ranging between 3–75 ms. Each acoustic pulse was modeled as a change in the excitatory and inhibitory conductance, governed by an alpha function with a 5 ms time constant (Fig. 2a, Wehr and Zador 2003). Our model consisted of three parameters: **1) I-E delay**- the temporal delay between inhibitory and excitatory inputs, **2) I/E ratio**- the ratio between the magnitude of inhibitory and excitatory inputs, and **3) Excitatory input**- the magnitude of the excitatory input (Fig. 2a). To ensure that the parameters of our model were physiologically realistic, we used a previously reported range of I/E ratio and I-E delay values obtained using intracellular recordings from auditory cortical neurons [24]. While our model does not explicitly simulate a specific cell-type or lamina of auditory cortex, the pattern of excitation combined with feedforward inhibition is consistent with the canonical circuitry of layer 2/3 auditory cortex, where synchronized and non-synchronized neurons have been previously identified in marmoset auditory cortex [14].

### Responses to pulse trains in real and simulated cortical neurons

We used two tests to classify neurons as synchronized or non-synchronized [14]. A synchronized neuron was required to have statistically significant vector strength at the longest IPI tested (Rayleigh statistic>13.8, P<0.001, at IPI = 75ms) [25]. Non-synchronized neurons were required to have a discharge rate ratio greater than one (i.e. the discharge rates at shorter IPIs had to be greater than at longer IPIs). If a neuron passed both of these criteria, it was classified as having a mixed response (S1 Fig). Mixed response neurons have been previously reported in auditory cortex [14], but at significantly lower proportions than either synchronized or non-synchronized neurons. If a neuron did not pass either criterion, it was classified as having an “atypical” response. In addition to these criteria, we only included neurons in our analysis with pure tone evoked discharge rates in the range of 1 to 50 spk/s. Our lower bound of 1 spk/s was to prevent the inclusion of neurons that were unresponsive. Our upper bound of 50 spk/s was to only include neurons that had discharge rates representative of a typical auditory cortical neuron [26] and avoid physiologically unrealistic responses. Using these criteria, we observed that the vast majority of neurons (98%) generated by our model could be classified as having a synchronized, non-synchronized, or mixed response (Fig. 2b).

In order to directly compare our computational model with real data, we reanalyzed a previously published dataset [15, 18, 26], composed of single-unit responses to acoustic pulse trains from the auditory cortex of four awake marmosets (*Callithrix jacchus*) (see Methods). Using the same criteria as in our computational model, 70% of units responding to our acoustic pulse train stimuli (147/210 units) could be classified as having synchronized, non-synchronized or a mixed response (Fig. 1, S1 Fig). The remaining neurons were more heterogeneous in their response properties; generally being weakly stimulus synchronized (but not at an IPI of 75 ms) and/or having an excited or suppressed response over a range of IPIs, with a tuning curve that was bandpassed, all passed, or high passed (only IPIs in the range of flutter perception). We also observed some of these less common response types in our model, including bandpassed and inhibitory (S2 Fig).

Next we compared the rate and temporal representations generated by real and simulated neurons. We observed that synchronized neurons, both real and simulated, were able to represent long IPIs using temporally locked responses (Fig. 2c), although simulated neurons had a substantially better temporal fidelity. Non-synchronized neurons, both real and simulated, represented shorter IPIs using a rate code (Fig. 2d), in which their normalized discharge rate decreased monotonically between IPIs in the range of 3 and 25 ms (i.e. higher discharge rates at shorter IPIs). Thus, the general features of temporal and rate representations produced by synchronized and non-synchronized neurons, respectively, were preserved in our computational model.

### Model parameters underlying rate and temporal representations

**Fig 3:**
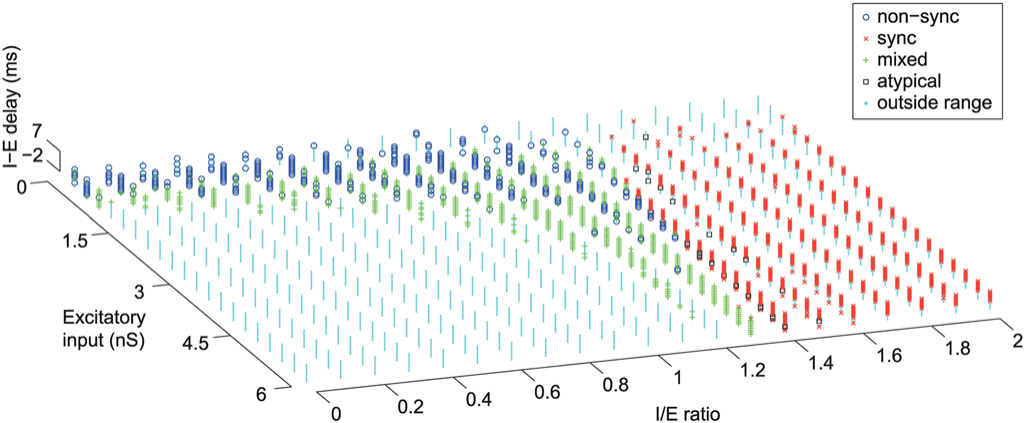
Dependence on input parameters of computational model. Classification of neuron-type [non-sync (o), sync (x), mixed (+), atypical (square)] across all three parameters (I-E delay, Excitatory input, and I/E ratio). If pure tone responses were less than 1 spk/s or greater than 50 spk/s, neurons were considered to have responses outside the allowable range (cyan) and were not included in our analysis.

What determines whether a neuron encodes an acoustic pulse train using a temporal and/or a rate representation? We observed that synchronized, non-synchronized, and mixed responses were generated within three distinct regions of the model’s parameter space (Fig. 3).

**Fig 4:**
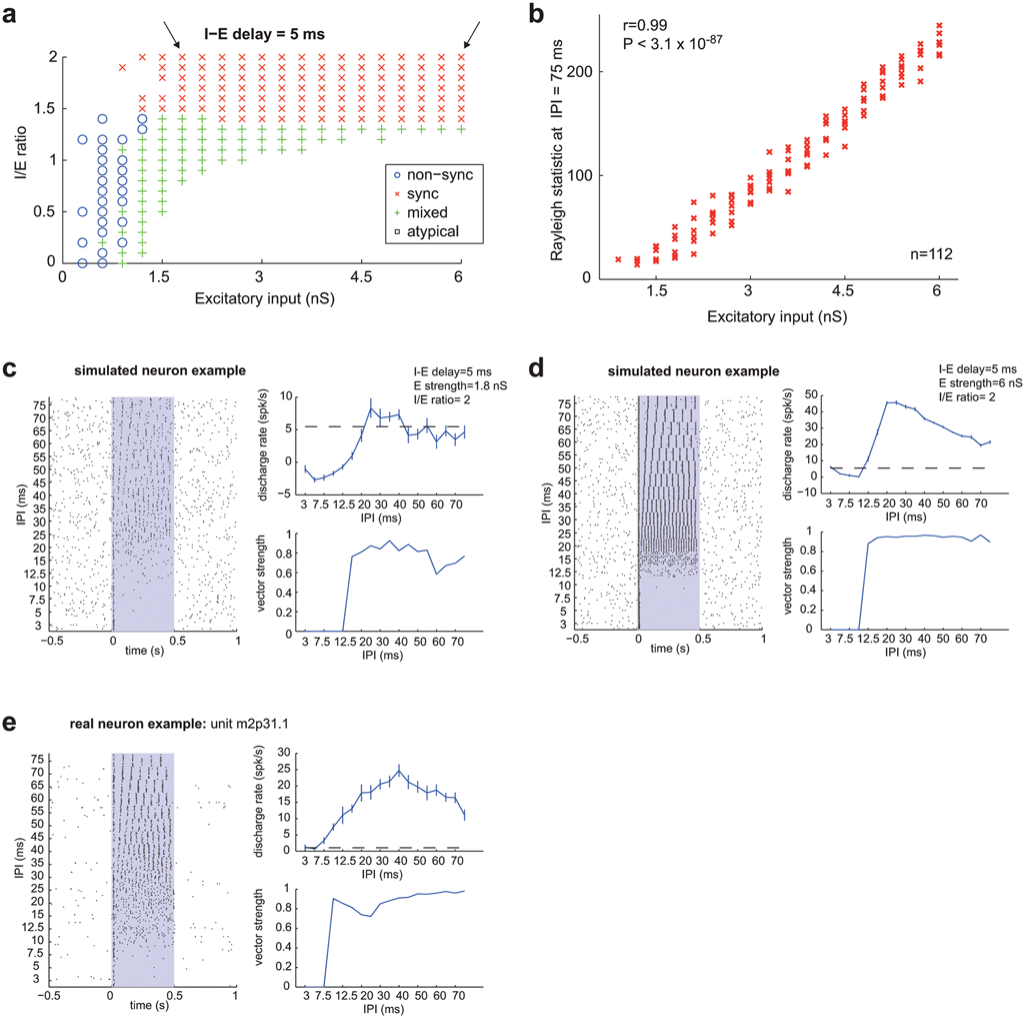
Simulated synchronized neuron. a. Classification of neuron-type [non-sync (o), sync (x), mixed (+), atypical (square)] across two parameters (Excitatory input and I/E ratio), with a fixed I-E delay of 5 ms. The two arrows indicate the parameters used for the simulated neurons in Fig. 4c (left arrow) and Fig. 4d (right arrow). b. Dependence of Rayleigh statistic (at an IPI of 75 ms) on the amplitude of excitatory inputs in simulated synchronized neurons. Spearman correlation coefficient: r = 0.99, P<3.1x10^-87^. c-e. Examples of simulated and real *synchronized* neurons. Each plot is subdivided into a raster plot (left), IPI vs discharge rate plot (top right), and IPI vs vector strength plot (bottom right). The stimulus is played for 500 ms, which is indicated with the gray rectangle in the raster plot. The dashed line in the IPI vs discharge rate plot indicates a significant evoked response above the spontaneous rate (2σ). Error bars indicate SEM. c. Simulated neuron: I-E delay = 5 ms, E strength = 1.8 nS, I/E ratio = 2. d. Simulated neuron: I-E delay = 5 ms, E strength = 6 nS, I/E ratio = 2. e. Real neuron (unit m2p31.1) from awake marmoset auditory cortex.

Synchronized neurons were more common when inhibition lagged excitation, and the magnitude of inhibition was at least 40–50% stronger than the magnitude of excitation (Fig. 4a, model with an I-E delay = 5 ms). Comparing synchronized neurons with a fixed I-E delay of 5 ms, we observed a highly significant correlation (r = 0.99, P<3.1x10^-87^, Spearman Correlation, Fig. 4b) between the excitatory input strength and the Rayleigh statistic, the criterion we used to measure the statistical significance of stimulus-synchronization. In other words, as the strength of excitation increased, our confidence in the high fidelity of a synchronized neuron’s temporal representation improved. This can be observed by comparing the responses of two simulated synchronized neurons that differ in their excitation strength parameter (Fig. 4c-d). While both of these examples of simulated neurons differ in the robustness of their temporal representation, they closely match the general properties of real synchronized neurons (Fig. 4e).

**Fig 5:**
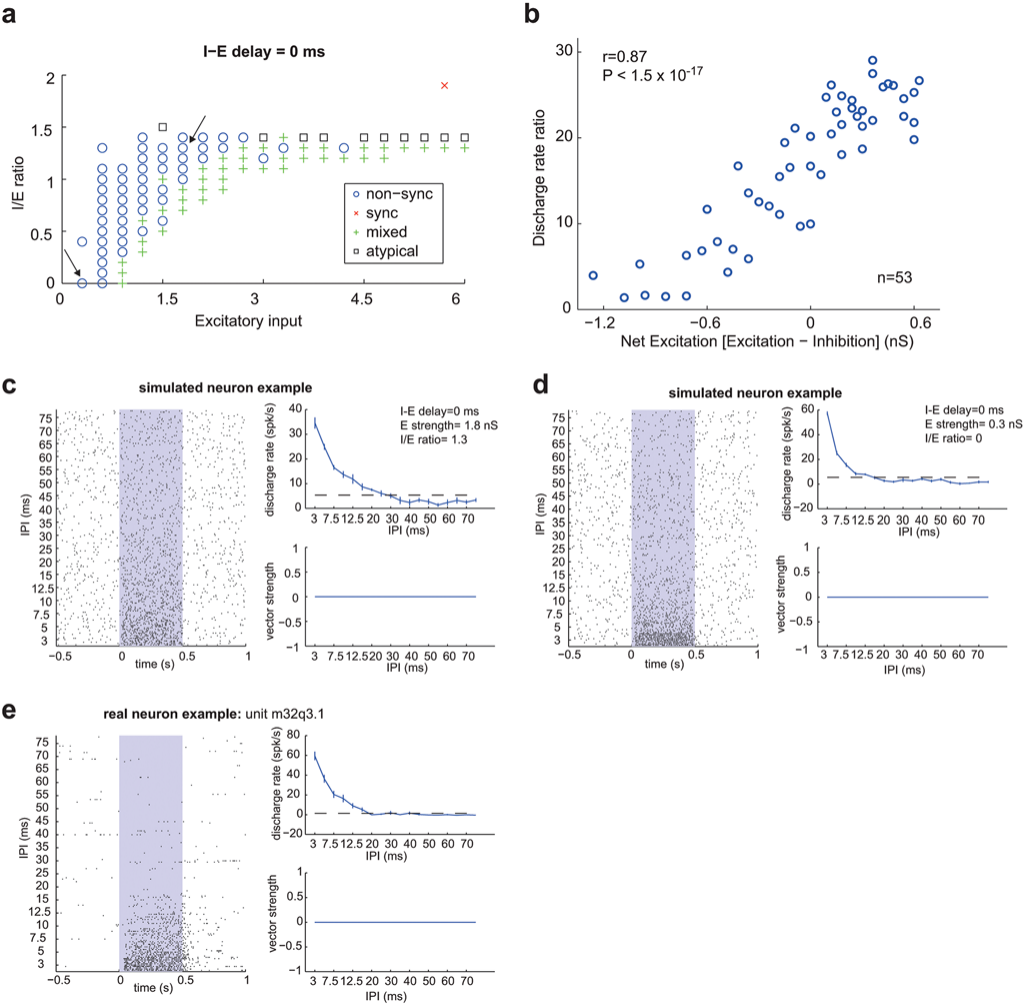
Simulated non-synchronized neuron. a. Classification of neuron-type [non-sync (o), sync (x), mixed (+), atypical (square)] across two parameters (Excitatory input and I/E ratio), with a fixed I-E delay of 0 ms. The two arrows indicate the parameters used for the simulated neurons in Fig. 5c (right arrow) and Fig. 5d (left arrow). b. Dependence of discharge rate ratio on net excitatory input (excitation-inhibition). Spearman correlation coefficient: r = 0.87, P<1.5 x 10^-17^ in simulated non-synchronized neurons. c-e. Examples of simulated and real *non-synchronized* neurons. Each plot is subdivided into a raster plot (left), IPI vs discharge rate plot (top right), and IPI vs vector strength plot (bottom right). The stimulus is played for 500 ms, which is indicated with the gray rectangle in the raster plot. The dashed line in the IPI vs discharge rate plot indicates a significant evoked response above the spontaneous rate (2σ). Error bars indicate SEM. c. Simulated neuron: I-E delay = 0 ms, E strength = 1.8 nS, I/E ratio = 1.3. d. Simulated neuron: I-E delay = 0 ms, E strength = 0.3 nS, I/E ratio = 0. e. Real neuron: (unit m32q3.1) from awake marmoset auditory cortex.

In contrast to this, non-synchronized neurons were more common when the net excitation was weak, which occurred for I/E ratios close to one (balanced excitation and inhibition) or low I/E ratios in combination with a weak excitatory input (Fig. 5a, model with an I-E delay = 0 ms). We observed a statistically significant correlation (r = 0.87, P<1.5 x 10^-17^, Spearman Correlation, Fig. 5b) between the net excitatory input (excitation-inhibition) and the discharge rate ratio. In other words, as the magnitude of net excitation increased, short IPIs evoked a higher discharge rate relative to the discharge rate at longer IPIs, effectively increasing the dynamic range of the neuron’s rate code. This effect can be observed by comparing the responses of two simulated non-synchronizing neurons that differ in their net excitation (Fig. 5c-d). While both of these examples of simulated neurons differ in the dynamic range of their rate representation, they closely match the general properties of real non-synchronized neurons (Fig. 5e).

When the net excitation increased above a conductance of approximately 0.6 nS, non-synchronized responses generally became mixed responses (Fig. 3, S1 Fig). Mixed responses could occur when the excitatory input of a non-synchronized neuron increased in strength, or alternatively when the inhibitory input of a synchronized neuron decreased in strength. In our computational model, roughly equal proportions of synchronized, non-synchronized, and mixed response neurons were generated (Fig. 3). This differs from our single-unit recordings, for which mixed response neurons (10%, 14/147 neurons) were less frequently observed than either synchronized or non-synchronized neurons. Because each parameter was uniformly distributed in our model, our results map the possible response types that can be generated, but not their relative proportions. For example, we allowed simulated neurons to have pure tone evoked discharge rates in the range of 1–50 spk/s, reflecting the range of discharge rates observed in our real data. However while evoked discharge rates can reach more than 50 spk/s in auditory cortex, lower discharge rates are more typical (median discharge rate = 16.4 spk/s). Reflecting this, we observed that when our criteria for a physiologically realistic response was lowered from 50 spk/s to 20 spk/s, the proportion of mixed response neurons decreased to about 12%, matching the proportion found in our single-unit recordings (S3 Fig).

### Comparison of model-based predictions and real neuronal responses

**Fig 6:**
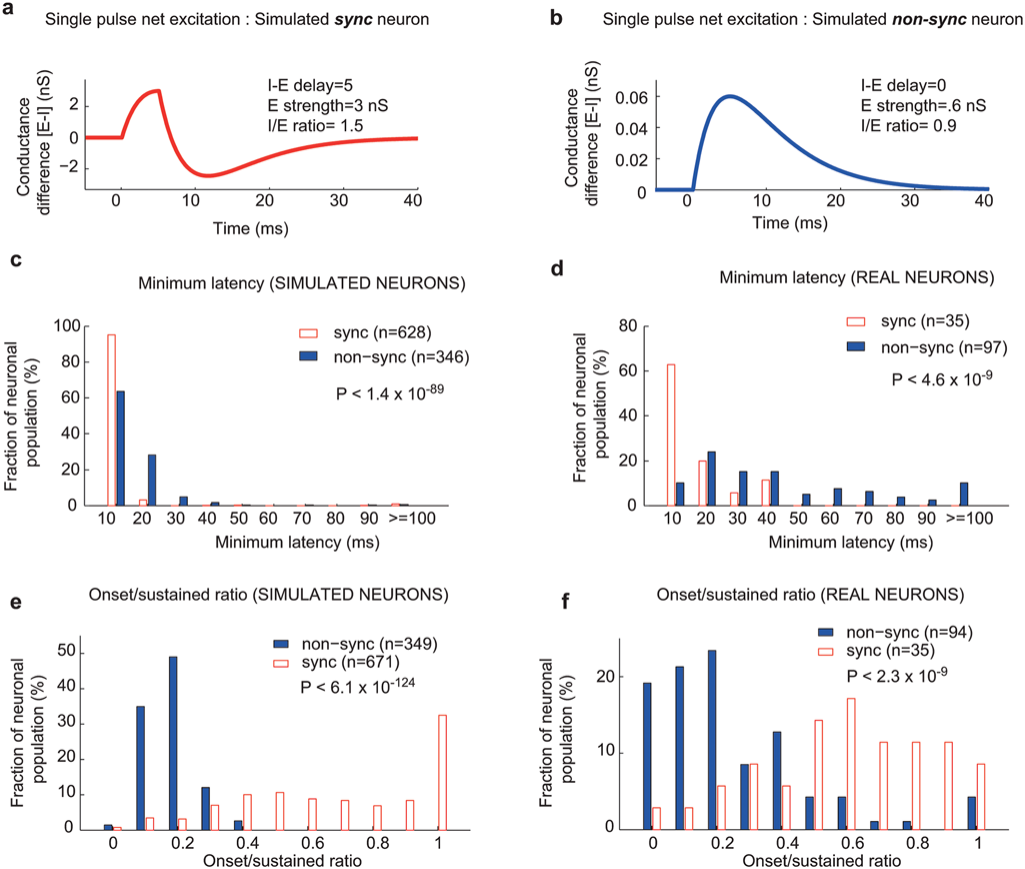
Temporal dynamics of synchronized and non-synchronized neurons. a. Net excitation (excitatory-inhibitory conductance) for a single acoustic pulse in a simulated *synchronized* neuron. I-E delay = 5 ms, E strength = 3 nS, I/E ratio = 1.5. b. Net excitation (excitatory-inhibitory conductance) for a single acoustic pulse in a simulated *non-synchronized* neuron. I-E delay = 0 ms, E strength = 0.6 nS, I/E ratio = 0.9. c. *Minimum latency* distribution for acoustic pulse train responses in *simulated* neurons. Mean: sync = 10.8 ms, nonsync = 16.6 ms, Wilcoxon rank sum test: P < 1.4 x 10^-89^. d. *Minimum latency* distribution for acoustic pulse train responses in *real* neurons. Mean: sync = 18.1 ms, nonsync = 51.1 ms, Wilcoxon rank sum test: P<4.6 x 10^-9^. e. *Onset/sustained ratio* distribution for pure tone responses in *simulated* neurons. Mean: sync = 0.69, nonsync = 0.18, Wilcoxon rank sum test: P < 6.1 x 10^-124^. f. *Onset/sustained ratio* distribution for pure tone responses in *real* neurons. Mean: sync = 0.60, nonsync = 0.25, Wilcoxon rank sum test: P<2.3 x 10^-9^.

Using our computational model, we could make several predictions concerning how stimulus-evoked responses may differ between synchronized, non-synchronized, and mixed-response neurons, which were testable in our dataset of real neurons. In our model, delayed inhibition in synchronized neurons led to a positive net excitation concentrated at the onset of the synaptic input (Fig. 6a), while balanced excitation and inhibition in non-synchronized neurons led to weaker net excitation that was spread out over a longer time duration (Fig. 6b). Because of this difference, an evoked response from a synchronized neuron only required a single acoustic pulse, while the evoked response of a non-synchronized neuron required the temporal summation of inputs from multiple acoustic pulses. We reasoned that this difference should also be reflected in the temporal dynamics of the neuron’s response to acoustic pulse trains, with a synchronizing neuron having a shorter latency to the onset of its response (minimum latency) than a non-synchronizing neuron. We measured the minimum latency of acoustic pulse train responses (see Methods), and found a statistically significant difference between synchronized and non-synchronized neurons, within both our simulated and real neuronal populations (Fig. 6c,d). In the *simulated* neuronal population, synchronized neurons had a mean minimum latency of 10.8 ms, while non-synchronized neurons had a mean minimum latency of 16.6 ms (Wilcoxon rank sum test, P < 1.4 x 10^-89^). A similar difference occurred in the *real* neuronal population; synchronized neurons had a mean minimum latency of 18.1 ms, while non-synchronized neurons had a mean minimum latency of 51.1 ms (Wilcoxon rank sum test, P < 4.6 x 10^-9^). Like synchronized neurons, mixed response neurons are also capable of envelope locking, with only a single acoustic pulse required to evoke a response. Based on this similarity, we reasoned that the minimum latency should be similar between synchronized and mixed response. We observed that mixed neurons had a mean minimum latency (simulated neurons: 8.0 ms, real neurons: 16.2 ms) not significantly different from synchronized neurons (Wilcoxon rank sum test, P = 0.053 (simulated), P = 0.30 (real)) and significantly different from non-synchronized neurons (Wilcoxon rank sum test, P<3.1x10^-75^ (simulated), P<1.1x10^-5^ (real)).

Using a similar reasoning, we hypothesized that the temporal dynamics of pure- tone responses should also differ between synchronized and non-synchronized neurons. Excitation concentrated at the onset of the synaptic input in synchronized neurons should evoke an onset response, while net excitation spread out over a longer time period in non-synchronized neurons should produce a more sustained response. To examine this we calculated the onset/sustained ratio to pure tones in our population of simulated and real neurons (see Methods), where a value of 1 indicated a pure onset response and a value of 0.25 indicated a sustained response. We observed a statistically significant difference between synchronized and non-synchronized responses in both simulated and real neurons (Fig. 6e,f). In the *simulated* neuronal population, synchronized neurons had a mean onset/sustained ratio of 0.69, while non-synchronized neurons had a mean onset/sustained ratio of 0.18 (Wilcoxon rank sum test, P < 6.1 x 10^-124^). A similar difference occurred in the *real* neuronal population; synchronized neurons had a mean onset/sustained ratio of 0.60, while non-synchronized neurons had a mean onset/sustained ratio of 0.25 (Wilcoxon rank sum test, P < 2.3 x 10^-9^). Thus, in both our real and simulated neuronal populations, synchronized neurons tended to have onset responses to pure tones, while non-synchronized neurons typically had sustained responses. This difference was not due to a slightly longer latency response in non-synchronizing neurons, as we also observed a statistically significant difference between synchronized and non-synchronized neurons when the time window used to calculate the onset discharge rate was lengthened to 100 ms (simulated neurons: P<1.61 x 10^-115^, real neurons: P<2.3x10^-5^, Wilcoxon rank sum test, see Methods).

These data suggest that the temporal dynamics of a pure tone response (onset or sustained) can be used to predict whether a neuron has a synchronized or non-synchronized response to an acoustic pulse train. However, some neurons can change between an onset and a sustained response when a sound’s acoustic parameters are altered; a sustained response is generally evoked by a preferred stimulus (best frequency and sound level) while an onset response can be generated by non-preferred stimuli [27]. Thus a neuron that has a non-synchronized response at its preferred sound level could theoretically switch to generate a synchronized response, when the sound level is increased above its preferred level. This can be observed in an example real neuron (see S4 Fig). When the sound level of a pure tone (at the neuron’s best frequency) was varied, this neuron responded with a sustained response at 10 dB SPL, and with an onset response at 70 dB SPL, causing a shift in the onset/sustained ratio from 0.4 to 0.8 respectively (S4 Fig, a-b). In response to acoustic pulse trains, this same neuron produced a non-synchronized response at 10 dB SPL, and was able to synchronize to acoustic pulse trains with IPIs above 20 ms when the sound level was elevated to 70 dB SPL (S4 Fig, c-d). The ability to switch between different neural coding regimes was observed in approximately 44% of neurons (8/18) tested with multiple acoustic pulse trains differing in sound level, frequency, or pulse width.

**Fig 7:**
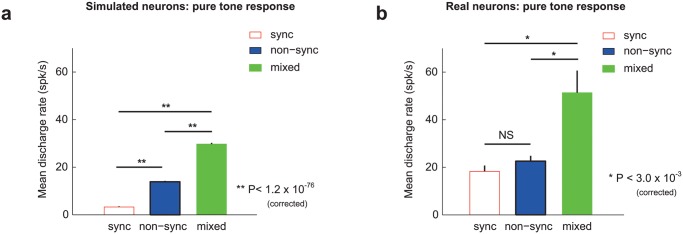
Discharge rates across mixed, synchronized, and non-synchronized neurons. Mean discharge rates for simulated (left) and real (right) neuronal populations, grouped according to their neural coding regime: synchronized (red), non-synchronized (blue), and mixed (green). Error bars indicate SEM. Wilcoxon rank sum test: * P<0.003 Bonferonni corrected, ** P<1.2x10^-76^ Bonferonni corrected, NS = not significant (P>0.05 uncorrected). a. Pure tone responses of simulated neurons. b. Pure tone responses of real neurons.

According to our computational model (Fig. 3), when either a synchronized neuron’s inhibitory input was reduced or a non-synchronized neuron’s excitatory input was increased, the neural coding regime changed to a mixed response (i.e. non-synchronized for short IPIs and synchronized for long IPIs). This implies that mixed response neurons had a larger net excitation than either synchronized or non-synchronized neurons, which we reasoned should manifest as a larger pure tone evoked response. We observed that for the simulated neuronal population, mixed neurons had a significantly higher discharge rate to pure tones than either non-synchronized and synchronized neurons (Fig. 7a, mixed = 29.7 spk/s, nonsync = 13.9 spk/s, sync = 3.3 spk/s; Wilcoxon rank sum test, P< 1.2 x 10^-76^, Bonferroni corrected). We also observed a significant difference between non-synchronized and synchronized neurons (Wilcoxon rank sum test, P< 6.9 x 10^-96^, Bonferroni corrected). We found a similar effect in our real neuronal population- mixed neurons had a significantly higher discharge rate to pure tones than either synchronized or non-synchronized neurons (Fig. 7b, mixed = 51.3 spk/s, nonsync = 22.5 spk/s, sync = 18.3 spk/s; Wilcoxon rank sum test, P< 0.003, Bonferroni corrected). While non-synchronized neurons had a slightly higher pure tone evoked response to pure tones than synchronized neurons, this difference was not statistically significant (Wilcoxon rank sum test, P = 0.58, uncorrected). Compared with real neurons, simulated synchronized neurons generally had lower firing rates. One potential reason for this is a higher percentage of simulated neurons receiving very strong inhibition than in our real neuronal population. However, we still observed both onset and sustained pure tone responses in simulated neurons, representative of typical real synchronizing neurons. Strong delayed inhibition (e.g. an I/E ratios of 2) typically generated onset responses with delayed suppression, while more moderate delayed inhibition (e.g. an I/E ratio of 1.4) typically generated onset responses with sustained activity (S5 Fig).

**Fig 8:**
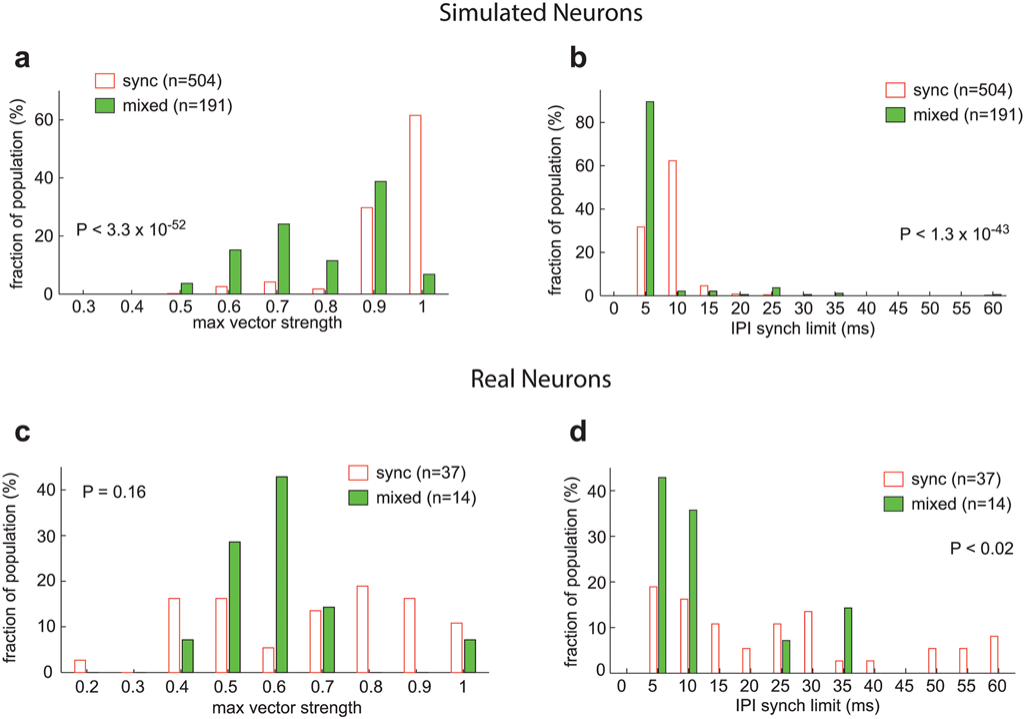
Temporal fidelity of synchronized and mixed neurons. Only simulated neurons with an excitatory input strength between 3–6 nS were used in this analysis, such that synchronized and mixed neurons had a similar distribution of excitatory levels. a. *Max vector strength* distribution for acoustic pulse train responses in *simulated* neurons. Mean: sync = 0.93, mixed = 0.79, Wilcoxon rank sum test: P < 3.3 x 10^-52^. b. *IPI synchronization limit* distribution for acoustic pulse train responses in *simulated* neurons. Mean: sync = 10.2 ms, mixed = 7.7 ms, Wilcoxon rank sum test: P < 1.3 x 10^-43^. c. *Max vector strength* distribution for acoustic pulse train responses in *real* neurons. Mean: sync = 0.68, mixed = 0.60, Wilcoxon rank sum test: P = 0.16. d. *IPI synchronization limit* distribution for acoustic pulse train responses in *real* neurons. Mean: sync = 25.7 ms, mixed = 13.4 ms, Wilcoxon rank sum test: P < 0.02.

We also hypothesized that the strength of a neuron’s inhibitory input would impact its temporal fidelity. Delayed inhibition suppresses random spiking that can occur between acoustic pulses; the resulting stimulus-locked response has a higher vector strength, thus providing a better temporal representation of the acoustic pulse train. As synchronized neurons have stronger delayed inhibition than mixed response neurons, synchronized neurons should therefore have higher vector strengths. However, this comes at a cost, as delayed inhibition prevents the response to a second acoustic pulse during the brief period that the neuron is suppressed. Thus mixed response neurons (which have less inhibition) should be able to stimulus synchronize at shorter IPIs than synchronized neurons. As predicted, we observed that for our simulated neuronal population, synchronized neurons had higher maximum vector strengths than mixed response neurons (Fig. 8a, sync = 0.93, mixed = 0.79; Wilcoxon rank sum test, P< 3.3 x 10^-52^, see Methods), while mixed response neurons had lower stimulus synchronization limits (Fig. 8b, sync = 10.2 ms, mixed = 7.7 ms; Wilcoxon rank sum test, P< 1.3 x 10^-43^). We observed a similar trend for our real neuron population- synchronized neurons had higher maximum vector strengths than mixed response neurons (Fig. 8c, sync = 0.68, mixed = 0.60; Wilcoxon rank sum test, P = 0.16), while mixed response neurons had lower stimulus synchronization limits (Fig. 8d, sync = 25.7 ms, mixed = 13.4 ms; Wilcoxon rank sum test, P< 0.02). Although we only observed a statistically significant difference between synchronized and mixed response neurons for the stimulus synchronization limit and not maximum vector strength, this may be due to the limited number of mixed response neurons that we were able to record from (n = 14). These results suggest that blocking inhibition (e.g. by adding a GABA-A antagonist such as Gabazine [28]), effectively decreasing the I/E ratio, should decrease a neuron’s vector strength while increasing its stimulus synchronization limit. However, the relationship between inhibition and temporal fidelity is more complex. While for simulated neurons with the same excitatory input strength, the stimulus synchronization limit of synchronized neurons decreased as the I/E ratio increased (P<0.001, Spearman correlation coefficient), we did not observe a statistically significant trend between the stimulus synchronization limit and I/E ratio in mixed response neurons (P>0.05, Spearman correlation coefficient).

In our computational model, the time-varying conductance used to simulate the neuron’s synaptic input was simplified to only approximate the AMPA and GABA-A currents evoked by the acoustic pulse train, with a time-constant of 5 ms [24] (see Methods). The synchronization limit of simulated neurons was also sensitive to this time constant; increasing this time constant (S6 Fig, a-d) or adding an additional NMDA-based conductance (S6 Fig (e-f) [29], see Methods) shifted the synchronization limit of simulated neurons to longer IPIs (mean synchronization limit: sync = 15.9 ms, mixed = 15.3 ms).

### Impact of spontaneous rate on computational model

**Fig 9:**
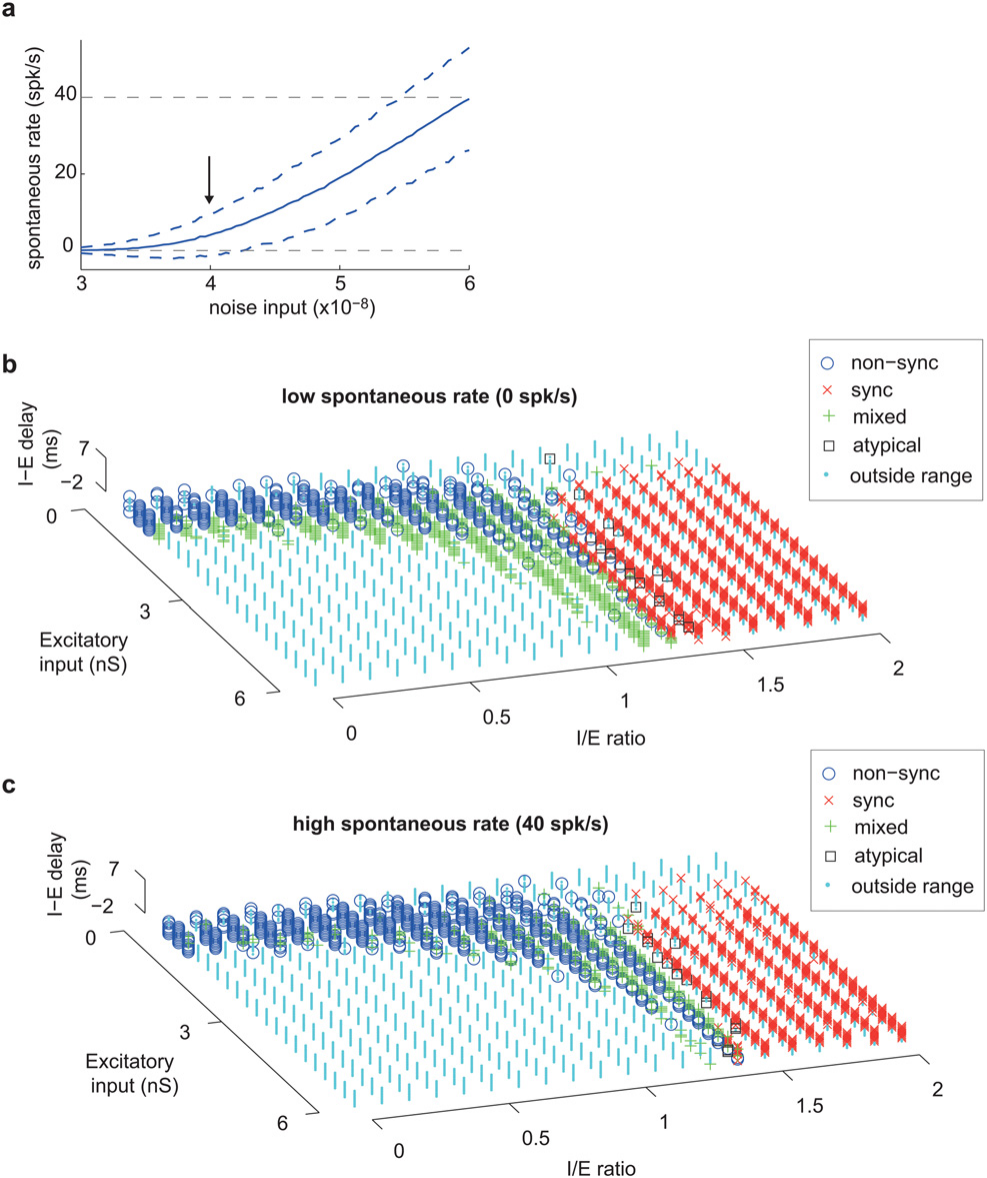
Impact of spontaneous rate on computational model. a. Relationship between spontaneous rate of simulated neuron and the amplitude of the Gaussian noise added to the excitatory and inhibitory conductances. The arrow indicates the amplitude of noise used for the simulated neurons in analyses conducted in Figs. 2–8. The gray dashed lines indicate spontaneous rates of 0 spk/s (bottom) and 40 spk/s (top). b-c. Classification of neuron-type [non-sync (o), sync (x), mixed (+), atypical (square)] for two different spontaneous rates: a low spontaneous rate (b) of 0 spk/s (noise input of 3x10^-8^) and a high spontaneous rate (c) of ~40 spk/s (noise input of 6x10^-8^). Responses outside the allowable range (pure tone response between 1–50 spk/s) are indicated in cyan.

**Fig 10:**
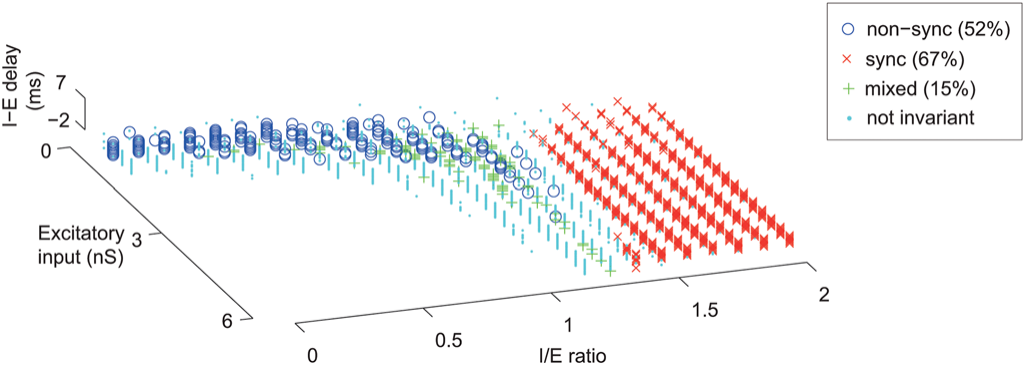
Invariance of response type across varying spontaneous rates. Response type invariance across varying spontaneous rates (between 0–40 spk/s). Model parameters yielding one response type across all spontaneous rates tested are indicated [non-sync (o), sync (x), mixed (+)]. Model parameters where neurons changed between response types (e.g. sync → non-sync) are shown in cyan.

Our computational model operated with a fixed spontaneous rate (~4 spk/s), close to the median spontaneous rate encountered in our real neuronal population (3.8 spk/s). To generate a spontaneous rate, we added Gaussian noise to the excitatory and inhibitory conductances of the neuron. If the amplitude of this Gaussian noise was increased, the spontaneous rate increased monotonically (Fig. 9a). We next examined how sensitive our computational model was to changes in spontaneous rate. We examined spontaneous rates covering the entire range observed in our real neuronal population (0–40 spk/s) and found that the model parameters for generating synchronized and non-synchronized neurons were similar, albeit with a slight shift in the threshold I/E ratio for observing synchronized responses (Fig. 9b,c). We next examined how robust each neuronal representation (sync, non-sync, mixed) was across varying spontaneous rates (0–40 spk/s), and observed that a large fraction of synchronized (67%) and non-synchronized (52%) neurons did not change their neural coding regime across the entire range of spontaneous rates tested (Fig. 10, S7 Fig). In contrast to this, only 15% of mixed response neurons showed a similar invariance (Fig. 10).

The addition of internal neuronal noise (see Methods), which generated the neuron’s spontaneous rate, was critical for our computational model. Removing this noise from our model almost completely eliminated non-synchronized responses, although mixed and synchronized responses were still preserved (S8 Fig). Furthermore, the non-synchronized responses generated in the absence of added noise had the unusual behavior of stimulus synchronizing at short IPIs, which was not a property of non-synchronized responses in our real and simulated neuronal populations (S8 Fig). Increasing the temporal jitter of synaptic inputs (S9 Fig) generated non-synchronized responses more typical of real neurons, while maintaining synchronized responses. While our model’s ability to generate non-synchronized responses required a source of internal noise (or sufficient temporal jitter of synaptic inputs), other methods of generating internal noise also produced similar results, including injecting noise as a current into the integrate-and-fire model to simulate background synaptic activity [30] (S10 Fig) and adding Gaussian noise to the membrane potential spiking threshold [31] (S11 Fig).

## Discussion

Temporal information within an acoustic signal can be represented in auditory cortex by a *temporal representation* of envelope-locked responses and/or a *rate representation* where discharge rate varies with interpulse interval [32]. Here we propose that the timing and magnitude of inhibition relative to excitation determines whether a neuron uses a temporal and/or rate representation. Using a computational model of an auditory cortical neuron, we found that synchronized responses were generated when strong inhibition lagged excitation. This created a stimulus-locked response at long interpulse intervals (IPIs), but an onset response followed by suppression at short IPIs. Conversely, non-synchronized responses were generated when excitation and inhibition were concurrent and balanced, which resulted in weak net excitation. This produced an input that was too weak to generate a response to a single acoustic pulse, but for sufficiently short IPIs (such that two or more pulses occurred within the neuron’s temporal integration window), the net excitation was sufficient to evoke a response. As the IPI duration was shortened further, more acoustic pulses occurred within the neuron’s temporal integration window and evoked a higher discharge rate, in turn creating a monotonic relationship between discharge rate and decreasing IPI. Thus non-synchronized neurons act as “integrators”, while the envelope-locked responses of synchronized neurons behave as “differentiators”. It is important to note that although non-synchronized neurons encode temporal rates in the range of pitch perception [33–35], they are insensitive to periodicity, and thus do not encode pitch salience [18] and may instead be generating a sensation more akin to roughness perception [36].

The parameters of our computational model were based on previously reported intracellular data from rodent auditory cortex [24], and consistent with intracellular data reported by other laboratories [37–41]. Based on the relationship between excitation and inhibition used to generate temporal and rate representations in our computational model, we were able to make several testable predictions including differences in the temporal fidelity, discharge rates and temporal dynamics of evoked responses, which were subsequently confirmed in our real neuronal population. One important observation supporting our model was that changes in the timing and strength of inhibition, occurring when sound level or frequency were altered [40], could shift a real neuron’s response between synchronized and non-synchronized (or vice-versa). While this indicates that a neuron’s neural code is not fixed, it is important to note that other potential mechanisms, such as synaptic facilitation and depression [42–44], recurrent connections [45–46], lateral connections [30,47], dendritic computation [48], or other non-linear neuronal properties [49] could also potentially contribute to the generation of synchronized and non-synchronized responses, and are not directly ruled out by our model. Using synaptic depression and facilitation, Rabang and Bartlett [50] have demonstrated an alternative method for generating temporal and rate representations; large inputs with synaptic depression create synchronized responses while weak inputs with mixed plasticity (synaptic depression of AMPA receptors and synaptic facilitation of NMDA receptors) generate mixed and non-synchronized responses. While synaptic facilitation and depression likely play a central role in temporal processing, we have demonstrated in our computational model that in the absence of synaptic facilitation and depression, feedforward inhibition is sufficient to generate many of the properties of temporal and rate representations observed in auditory cortex.

While non-synchronized responses have been reported in several different species (e.g. *monkey* [14,17–18], *cat* [19], and *rat* [51]), many previous studies have only reported stimulus-synchronized responses in auditory cortex. Our model provides several suggestions why this may be the case. First, many previous studies have used anesthetized animals to investigate temporal processing in auditory cortex. Because non-synchronized responses are generated by weak net-excitation, usually by concurrent excitation and inhibition, any anesthesia related decrease in excitation or increase in inhibition would further decrease the neuron’s net excitation, potentially silencing non-synchronized responses [52–54]. Because synchronized neurons have delayed inhibition, they would be less affected by anesthesia, as a large variety of I/E ratios and E strengths will still generate a synchronized response (Fig. 3). The second issue that may reduce the number of non-synchronized responses observed is related to stimulus optimization. For a given neuron, an optimal stimulus can produce a sustained response, while a non-optimal stimulus will often only produce an onset response [27], the latter likely resulting from delayed inhibition. As delayed inhibition will most likely generate a synchronized response, rather than non-synchronized, there may be a large bias towards observing synchronized response when the non-optimal sound level or frequency is used. The third issue is the choice of temporally modulated acoustic stimuli used by the experimenter. For example, when using sinusoidal amplitude modulated tones [26,55–56] the pulse duration changes with modulation frequency, in contrast to acoustic pulse trains that have fixed pulse durations. While non-synchronized neurons do not respond to single pulses at long IPIs for an acoustic pulse train, this would likely change when the pulse duration is sufficiently lengthened, providing enough excitatory drive for the neuron to spike and resulting in synchronization at low modulation frequencies.

Finally, it is important to acknowledge several caveats with our computational model. First, our study directly compares single units from awake marmosets with simulated neurons generated by a neuronal model that is based on intracellular recordings from ketamine-anesthetized rats [24]. Because ketamine is an NMDA antagonist, our model is based primarily on AMPA and GABA-A receptors. Using only synaptic inputs with a short time-constant (5 ms), approximating AMPA and GABA-A receptors, our model was able to simulate the major types of neural representations (synchronized, non-synchronized, and mixed), as well as two atypical types (inhibitory and bandpassed). We observed that adding NMDA receptors to our model,(see S6 Fig, e-f) shifted the synchronization boundary to longer IPIs, and closer to the synchronization limit observed in real synchronizing neurons. It’s important to point out that our model was not able to capture the complete diversity of responses observed in auditory cortex. Most notably, we did not observe unsynchronized responses in the flutter range, which have been previously reported in auditory cortex, albeit more commonly in the rostral and rostrotemporal fields [15–16]. These responses are similar to non-synchronized responses described here, except that the encoding range of IPIs is between 20–125 ms (perceptual range of flutter), rather than 3–25 ms (perceptual range of fusion), and that the relationship between IPI and discharge rate can be either positive or negative monotonic. Given that these responses are more common outside of primary auditory cortex, and have a longer latency response [15], a two-stage model may be required to generate this type of neural representation. Next, our model was only accurate in representing the neural responses observed in auditory cortex to acoustic pulse trains (Gaussian windowed tones), which have a fixed bandwidth over the complete range of IPIs tested. Other acoustic stimuli, such as sinusoidal amplitude modulated (sAM) tones, that change their spectral bandwidth and pulse duration with modulation frequency, cannot be represented accurately by our model, however the addition of new parameters that account for the spectrum of the acoustic stimulus [47] and/or the adaptation of synaptic inputs [42] would likely provide further improvements to our description of temporal processing in auditory cortical neurons.

In spite of these caveats, our model provides a parsimonious explanation for the neural coding dichotomy of synchronized and non-synchronized responses observed in auditory cortex. One key implication of these results is that if excitation and inhibition can be independently manipulated, which is possible using molecular genetic techniques such as optogenetics [57], a neuron’s neural representation can theoretically be switched between synchronized and non-synchronized. In a behaving animal, this provides a powerful paradigm to directly test the auditory percepts generated by temporal and rate-based neural representations.

## Methods

### Ethics statement

The electrophysiology data in this report comprised of previous published datasets [15,18,26] collected at Johns Hopkins University (Laboratory of Xiaoqin Wang). All experimental procedures were approved by the Johns Hopkins University Animal Use and Care Committee and followed US National Institutes of Health guidelines.

### Computational model

An integrate-and-fire computational neuronal model was simulated in MATLAB using the following equation, using parameters obtained from Wehr and Zador [24]:

$$V_{t+1}=-\frac{dt}{C}\left[g_{e_{t}}\left(V_{t}-E_{e}\right)+g_{i_{t}}\left(V_{t}-E_{i}\right)+g_{rest}\left(V_{t}-E_{rest}\right)\right]+V_{t}$$

with C = 0.25 nF and g_rest_ = 25 nS.

Each acoustic pulse was simulated as the summation of 10 excitatory and 10 inhibitory synaptic inputs [24], each temporally jittered (Gaussian distribution, σ = 1 ms). Each synaptic input was modeled as a time-varying conductance fit to an alpha function:

$$g\left(t\right)=Ate^{-at}$$

with a time constant 5 ms and an amplitude determined by the excitatory input parameter of the model (ranging from 0.3 to 6 nS). A 10 ms delay was added to the synaptic input to simulate the delay between peripheral auditory system and auditory cortex. The reversal potential for the excitatory and inhibitory inputs were 0 mV and -85 mV respectively. A timestep of 0.1 ms was used for the simulation.

Our model consisted of three parameters: 1) I-E delay- the temporal delay between inhibitory and excitatory inputs, 2) I/E ratio- the ratio between the magnitude of inhibitory and excitatory inputs, and 3) Excitatory input- the magnitude of the excitatory input. The first two parameters (I-E delay and I/E ratio) spanned the entire range of values measured intracellularly, previously reported by Wehr and Zador [24] [I-E delay: -2 to 7 ms, I/E ratio: 0 to 2]. Physiologically realistic values for excitation magnitude (3^rd^ parameter) [spanning values of 0.3 nS to 6 nS] were determined by calculating the discharge rate of simulated neurons to a pure tone stimulus. For a simulated neuron to be included in our analysis, we required an evoked pure tone response (greater than 1 spk/s) without having a response greater than 50 spk/s (to avoid physiologically uncommon or unrealistic levels of excitation). The only change to our model if extending this boundary to higher discharge rates would be the inclusion of more synchronized and mixed response neurons, with even higher evoked discharge rates.

Gaussian noise was added to the model using three methods: 1) noise added to the time-varying excitatory and inhibitory conductances to simulate random channel fluctuations, 2) noise added to the current to simulated background synaptic activity contributing to the spontaneous rate, or 3) noise added to the membrane potential based spiking threshold.

NMDA channels were added to the model only for S6 Fig (e-f.) The time constants used for the alpha function governing the time varying conductance for the NMDA channel was 63 ms (fast component) and 200 ms (slow component), with a peak amplitude ratio of 0.88:0.12 (fast:slow). The ratio of the peak amplitude between the AMPA channel (time constant of 5 ms) and NMDA channel was 1:0.3 (AMPA:NMDA) [29].

#### Pure tone responses

Pure tone stimuli were generated in the model by convolving a step function (duration of tone) with a single acoustic pulse. Pure tones were 200 ms in duration.

#### Generation of spontaneous rate

To generate a spontaneous rate, Gaussian noise was added to the excitatory and inhibitory conductances (μ = 0). In all analyses, unless otherwise noted, σ = 4x10^-8^. The range of σ used in all experiments was between 3x10^-8^ and 6x10^-8^ (see Fig. 9a). As an alternative method of generating a spontaneous rate, Gaussian noise (μ = 0, σ = 1 mV) was added as an injected current (S10 Fig) or Gaussian noise (μ = 0, σ = 3 mV) was added to the membrane potential spiking threshold, normally set at -45 mV (S11 Fig).

#### Discharge rate calculation

The mean spontaneous rate was subtracted from all calculations of the discharge rate for simulated and model neurons. The thresholds of >1 spk/s and <50 spk/s for simulated neurons includes the subtraction of the mean spontaneous rate.

### Electrophysiological recordings and acoustic stimuli

Our electrophysiology data in this report comprised of previous published datasets [15,18,26]. For these datasets, we performed single-unit recordings with high-impedance tungsten microelectrodes (2–5 MΩ) in the auditory cortex of four awake, semi-restrained common marmosets (*Callithrix jacchus*), a new-world primate species. Action potentials were sorted on-line using a template-matching method (MSD, Alpha Omega Engineering). Experiments were conducted in a double-walled, soundproof chamber (Industrial Acoustic Co., Inc.) with 3-inch acoustic absorption foams covering each inner wall (Sonex, Illbruck, Inc.). Acoustic stimuli were generated digitally (MATLAB- custom software, Tucker Davis Technologies) and delivered by a free-field speaker located 1 meter in front of the animal. This physiological data was collected at Johns Hopkins University (Laboratory of Xiaoqin Wang).

Recordings were made primarily for the three core fields of auditory cortex (177/210 neurons)- primary auditory cortex (AI), the Rostral field (R), and the Rostrotemporal field (RT), with the remaining neurons recorded from surrounding belt fields. For each single unit isolated, the best frequency (BF) and sound level threshold was first measured, using pure tone stimuli that were 200 ms in duration. We next generated a set of acoustic pulse trains, where each pulse was generated by windowing a brief tone at the BF by a Gaussian envelope. Interpulse intervals (IPIs) ranged from 3 ms to 75 ms (3, 5, 7.5, 10, 12.5, 15, 20, 25, 30, 35, 40, 45, 50, 55, 60, 65, 70, 75 ms). Acoustic pulse train stimuli were 500 ms in duration, and all intertrial intervals were at least 1 s long. Each stimulus was presented in a randomly shuffled order with other stimuli, and repeated at least five times for all neurons, and at least ten times for about 55% of neurons (115/210). Stimulus intensity levels for acoustic pulse trains were generally 10–30 dB above BF-tone thresholds for neurons with monotonic rate-level functions and at the preferred sound level for neurons with non-monotonic rate-level functions.

### Data analysis

#### Classification of neural representation

We used two tests to classify neurons as synchronized or non-synchronized [14]. A synchronized neuron was required to have statistically significant vector strength at the longest IPI tested (Rayleigh statistic>13.8, P<0.001, at IPI = 75ms). Non-synchronized neurons were required to have a discharge rate ratio greater than one (i.e. the discharge rates at shorter IPIs had to be greater than at longer IPIs). If a neuron passed both of these criteria, it was classified as having a mixed response. Conversely, if a neuron did not pass either criterion, it was classified as having an “atypical” response. Thus neurons could be classified as having a synchronized, non-synchronized, mixed, or atypical response type, or alternatively not be included in our analysis as a result of being unresponsive (<1 spk/s to pure tones) or having a physiologically unrealistic response (>50 spk/s to pure tones). Synchronized neurons were not required to have a pure tone response greater than 1 spk/s.

In our real neuronal population, neurons were considered to have significant response, and included for further analysis if they pass the criteria for synchronized, non-synchronized, mixed, or atypical response type. Neurons not classified as synchronized, non-synchronized, or mixed response, were only included in our analysis (as an atypical response) if they responded to acoustic pulse trains; the criteria for this was a significant vector strength for two neighboring IPIs and/or firing rate significantly above or below (2 σ) the spontaneous rate for two neighboring IPIs.

*Vector strength* was calculated using the following equation:

$$\text{VS}=\left(1/\text{n}\right)\sqrt{{\left(\text{sin}\left(\frac{2{\pi \text{t}}_{\text{i}}}{\text{IPI}}\right)\right)}^{2}+{\left(\text{cos}\left(\frac{2{\pi \text{t}}_{\text{i}}}{\text{IPI}}\right)\right)}^{2}}$$

Where VS = vector strength, n = number of spikes, t_i_ = time of i^th^ spikes (relative to previous acoustic pulse), and IPI = interpulse interval

For Fig. 8, vector strength and IPI synchronization limit were calculated for all simulated neurons with excitatory inputs between 3–6 nS. This restricted range was used so that our comparison between synchronized and mixed response neurons was based on a similar level of excitation.

*Rayleigh statistic* was calculated using the following equation: 2n(VS)^2^

where n = number of spikes, and VS = vector strength

Values of the Rayleigh statistic greater than 13.8 were considered statistically significant (P < 0.001) [25].

#### Discharge rate ratio

The firing rate at an IPI of 3 ms divided by the maximum firing rate for all IPIs in the range of 35 ms and 75 ms.

#### Minimum latency calculation

The minimum latency was the first 2 ms time bin where the discharge rate was greater than 3 σ above the spontaneous rate and at least 2 spikes had occurred [26]. The two successive time bins (2–4 ms, 4–6 ms) also were required to have discharge rates greater than 3 σ above the spontaneous rate. All evoked response to acoustic pulse trains with discharge rates greater than 2 σ above the spontaneous rate were pooled together and included in this calculation.

#### Onset/sustained ratio

this ratio was calculated as follows:

$$\text{Onset}/\text{sustained ratio}\;=\;{\text{S}}_{\left[0–50\;\text{ms}\right]}\;/\;{\text{S}}_{\left[0–200\;\text{ms}\right]},$$

where S_[t1-t2]_ is the number of spikes in the time window of t1 to t2 (relative to the onset of the pure tone). A value of 1 indicated an onset response (all spikes occurred within the first 50 ms), while a value of 0.25 indicated a sustained response.

## Supporting Information

S1 Fig Examples of mixed responses from real and simulated neurons. Each plot is subdivided into a raster plot (left), IPI vs discharge rate plot (top right), and IPI vs vector strength plot (bottom right). The stimulus is played for 500 ms, which is indicated with the gray rectangle in the raster plot. The dashed line in the IPI vs discharge rate plot indicates a significant evoked response above the spontaneous rate (2 σ). Error bars indicate SEM. a. *Mixed* response, *real* neuron. b. *Mixed* response, *simulated* neuron (I-E delay = 3 ms, E strength = 3.6 nS, I/E ratio = 1.3).
(EPS)

S2 Fig Examples atypical responses (bandpassed and inhibitory) from real and simulated neurons. Each plot is subdivided into a raster plot (left), IPI vs discharge rate plot (top right), and IPI vs vector strength plot (bottom right). The stimulus is played for 500 ms, which is indicated with the gray rectangle in the raster plot. The dashed line in the IPI vs discharge rate plot indicates a significant evoked response above the spontaneous rate (2 σ). Error bars indicate SEM. a. *Atypical* response (bandpassed), *real* neuron. b. *Atypical* response (bandpassed), *simulated* neuron (I-E delay = 0 ms, E strength = 3 nS, I/E ratio = 1.4). c. *Atypical* response (inhibitory), *real* neuron. d *Atypical* response (inhibitory), *simulated* neuron (I-E delay = 0 ms, E strength = 3 nS, I/E ratio = 5, noise input = 5x10^-8^, spont rate = 19 spk/s).
(EPS)

S3 Fig Effect of discharge rate criterion on mixed responses. a. Proportion of synchronized, non-synchronized, and mixed response neurons when the criterion for maximum discharge rate to a pure tone response is varied between 50 and 20 spikes/sec. b-c. Classification of neuron-type [non-sync (o), sync (x), mixed (+), atypical (square)] across two parameters (Excitatory input and I/E ratio), with a fixed I-E delay of (b) 0 ms and (c) 5 ms using a criterion of the maximum discharge rate to a pure tone being less than **20 spk/s**. d. Classification of neuron-type [non-sync (o), sync (x), mixed (+), atypical (square)] across all three parameters (I-E delay, Excitatory input, and I/E ratio) using a criterion of the maximum discharge rate to a pure tone being less than **20 spk/s**. If pure tone responses were less than 1 spk/s or greater than **20 spk/s**, neurons were considered to have responses outside the allowable range (cyan) and were not included in our analysis.
(EPS)

S4 Fig Example of a real neuron (unit m32q47.1) switching between synchronized and non-synchronized response modes. a. Raster plot of rate-level response (pure tone at neuron’s best frequency played at sound levels -10 to 70 dB SPL). Bolded values on y-axis indicate sound levels used for example 1 and example 2 in **b**. b. Dependence of onset/sustained ratio on sound level of pure tone. c-d. Each plot is subdivided into a raster plot (left), IPI vs discharge rate plot (top right), and IPI vs vector strength plot (bottom right). The stimulus is played for 500 ms, which is indicated with the gray rectangle in the raster plot. The dashed line in the IPI vs discharge rate plot indicates a significant evoked response above the spontaneous rate (2σ). Error bars indicate SEM. c. A *non-synchronized* response at a sound level of *10 dB SPL* (example 1). d.A *synchronized* response at a sound level of *70 dB SPL* (example 2).
(EPS)

S5 Fig Pure tone responses in simulated synchronized neurons. For each simulated neuron, the raster plot (above) and PSTH (below) are shown for a 200 ms duration pure tone played at time 0. a. Example onset response with delayed suppression: I-E Delay = 5 ms, Excitatory strength = 6 nS, I/E ratio = 2. b. Example onset response with sustained activity, I-E Delay = 5 ms, Excitatory strength = 2.4 nS, I/E ratio = 1.4.
(EPS)

S6 Fig Dependence of synchronization limit on conductance time constant. Simulated synchronizing neuron with I-E delay = 5 ms, excitatory strength = 3 nS, I/E ratio = 1.7. a. Raster plot of acoustic pulse train response, 5 ms time constant used for input conductance. b. Raster plot of acoustic pulse train response, 10 ms time constant used for input conductance. c. Raster plot of acoustic pulse train response, 20 ms time constant used for input conductance. d. Comparison of vector strength across three different time constants used for input conductance. e. Raster plot of acoustic pulse train response for a simulated neuron with both AMPA and NMDA currents. f. Vector strength for a simulated neuron with both AMPA and NMDA currents.
(EPS)

S7 Fig Examples of rate and temporal representations invariant to changes to the spontaneous rate. Each plot is subdivided into a raster plot (left), IPI vs discharge rate plot (top right), and IPI vs vector strength plot (bottom right). The stimulus is played for 500 ms, which is indicated with the gray rectangle in the raster plot. The dashed line in the IPI vs discharge rate plot indicates a significant evoked response above the spontaneous rate (2σ). Error bars indicate SEM. a. Example simulated *synchronized* neuron, *low* spontaneous rate (I-E delay = 5 ms, E strength = 3 nS, I/E ratio = 2, noise input = 3x10^-8^, spont rate<0.1 spk/s). b. Example simulated *synchronized* neuron, *high* spontaneous rate (I-E delay = 5 ms, E strength = 3 nS, I/E ratio = 2, noise input = 6x10^-8^, spont rate = 40.1 spk/s). c. Example simulated *non-synchronized* neuron, *low* spontaneous rate (I-E delay = 0 ms, E strength = 0.6 nS, I/E ratio = 0.3, noise input = 3x10^-8^, spont rate<0.1 spk/s). d. Example simulated *non-synchronized* neuron, *high* spontaneous rate (I-E delay = 0 ms, E strength = 0.6 nS, I/E ratio = 0.3, noise input = 6x10^-8^, spont rate = 39.4 spk/s).
(EPS)

S8 Fig Requirement of added neuronal noise for the generation of non-synchronized responses. a. Classification of neuron-type [non-sync (o), sync (x), mixed (+), atypical (square)] across all three parameters (I-E delay, Excitatory input, and I/E ratio) **using a noise input of 0 (spont rate = 0)**. If pure tone responses were less than 1 spk/s or greater than 50 spk/s, neurons were considered to have responses outside the allowable range (cyan) and were not included in our analysis. Non-synchronized responses were rare, and had the atypical behavior of synchronizing at short IPIs (see b). b. Example of an atypical non-synchronized response (vector strength not significant at an IPI of 75 ms) that is generated when the noise input is zero (spont rate = 0 spk/s). Although it is not a criterion for a non-synchronized response, the vector strength is typical not significant across all IPIs. However, for this example neuron, a significant vector strength is observed for IPIs between 10 and 15 ms. The plot is subdivided into a raster plot (left), IPI vs discharge rate plot (top right), and IPI vs vector strength plot (bottom right). The stimulus is played for 500 ms, which is indicated with the gray rectangle in the raster plot. The dashed line in the IPI vs discharge rate plot indicates a significant evoked response above the spontaneous rate (2σ). Error bars indicate SEM.
(EPS)

S9 Fig Addition of increased temporal jitter of inputs to computational model. Other than increased temporal jitter of synaptic inputs (uniform distribution, σ = 8.7 ms), no other sources of noise were added to the model. a. Classification of neuron-type [non-sync (o), sync (x), mixed (+), atypical (square)] across all three parameters (I-E delay, Excitatory input, and I/E ratio) using the temporal jitter of inputs as a source of noise. If pure tone responses were less than 1 spk/s or greater than 50 spk/s, neurons were considered to have responses outside the allowable range (cyan) and were not included in our analysis. b. Example of a non-synchronized response with identical parameters to the example neuron displayed in S8 Fig. The plot is subdivided into a raster plot (left), IPI vs discharge rate plot (top right), and IPI vs vector strength plot (bottom right). The stimulus is played for 500 ms, which is indicated with the gray rectangle in the raster plot. The dashed line in the IPI vs discharge rate plot indicates a significant evoked response above the spontaneous rate (2σ). Error bars indicate SEM.
(EPS)

S10 Fig Basic response types observed when Gaussian noise (μ = 0, σ = 1 mV) was added as an *input current* for a source of internal neuronal noise. a. Classification of neuron-type [non-sync (o), sync (x), mixed (+), atypical (square)] across all three parameters (I-E delay, Excitatory input, and I/E ratio) using a different method of adding noise to the model. Pure tone responses were less than 1 spk/s or greater than 50 spk/s, neurons were considered to have responses outside the allowable range (cyan) and were not included in our analysis. b. Example simulated synchronizing neuron: I-E delay = 5 ms, E strength = 3.6 nS, I/E ratio = 2. c. Example simulated non-synchronizing neuron: I-E delay = 0 ms, E strength = 0.3 nS, I/E ratio = 0.
(EPS)

S11 Fig Basic response types observed when Gaussian noise (μ = 0, σ = 3 mV) was added to the *membrane spiking threshold* for a source of internal neuronal noise. a. Classification of neuron-type [non-sync (o), sync (x), mixed (+), atypical (square)] across all three parameters (I-E delay, Excitatory input, and I/E ratio) using a different method of adding noise to the model. Pure tone responses were less than 1 spk/s or greater than 50 spk/s, neurons were considered to have responses outside the allowable range (cyan) and were not included in our analysis. b. Example simulated synchronizing neuron: I-E delay = 5 ms, E strength = 2.4 nS, I/E ratio = 2. c. Example simulated non-synchronizing neuron: I-E delay = 0 ms, E strength = 0.6 nS, I/E ratio = 0.5.
(EPS)
